# The Effect of a Duplex Surface Treatment on the Corrosion and Tribocorrosion Characteristics of Additively Manufactured Ti-6Al-4V

**DOI:** 10.3390/ma16052098

**Published:** 2023-03-04

**Authors:** Kelsey Ann Vella, Joseph Buhagiar, Glenn Cassar, Martina Marie Pizzuto, Luana Bonnici, Jian Chen, Xiyu Zhang, Zhiquan Huang, Ann Zammit

**Affiliations:** 1 Department of Metallurgy and Materials Engineering, University of Malta, MSD 2080 Msida, Malta; 2 School of Materials Science and Engineering, Southeast University, Nanjing 211189, China

**Keywords:** surface engineering, selective laser melting, tribocorrosion, corrosion, Ti6Al4V

## Abstract

The use of additively manufactured components specifically utilizing titanium alloys has seen rapid growth particularly in aerospace applications; however, the propensity for retained porosity, high(er) roughness finish, and detrimental tensile surface residual stresses are still a limiting factor curbing its expansion to other sectors such as maritime. The main aim of this investigation is to determine the effect of a duplex treatment, consisting of shot peening (SP) and a coating deposited by physical vapor deposition (PVD), to mitigate these issues and improve the surface characteristics of this material. In this study, the additive manufactured Ti-6Al-4V material was observed to have a tensile and yield strength comparable to its wrought counterpart. It also exhibited good impact performance undergoing mixed mode fracture. It was also observed that the SP and duplex treatments resulted in a 13% and 210% increase in hardness, respectively. Whilst the untreated and SP treated samples exhibited a similar tribocorrosion behavior, the duplex-treated sample exhibited the greatest resistance to corrosion-wear observed by the lack of damage on the surface and the diminished material loss rates. On the other hand, the surface treatments did not improve the corrosion performance of the Ti-6Al-4V substrate.

## 1. Introduction

Ti-6Al-4V is a versatile titanium alloy with an excellent combination of material characteristics making it exceedingly useful for a large range of applications including in the marine sector. Such properties include its excellent corrosion resistance in saline environments, high specific strength, ability of providing a significant reduction in weight and its relatively maintenance free nature when compared to other materials [1,2]. Some current marine applications include its use for the production of propellers and propeller shafts and for power and transmission equipment [2,3]. 

Such components are traditionally manufactured through conventional subtractive methods; thus, the first main objective of this investigation was to determine whether additive manufacturing can be a suitable replacement allowing for potential reduction in lead times and associated costs. Additive manufacturing allows the rapid near net shape production of components irrespective of their complexity. Components produced through such means require no or limited post processing and result in the production of minimal material waste [4,5]. 

However, additive manufacturing also presents its own limitations. Improper control over printing parameters can result in highly porous components and an associated poor surface finish. Furthermore, the high temperatures required to melt and fuse the metallic powder result in tensile residual stresses [6]. All of which can be very detrimental to the mechanical, corrosion, anti-fouling, and wear characteristics of the material under typical marine conditions. Thus, to combat this limitation—as well as that of the material itself, which is its poor behavior under friction conditions—a duplex surface treatment is proposed.

This treatment comprises of the dual application of shot peening and a coating deposited via physical vapor deposition. In shot peening, a stream of shots impinges the surface at room temperature, making it a cold work process. This impingement causes work hardening at the surface and induces compressive residual stresses, which are beneficial as they mitigate any crack initiation and propagation [7]. The shot peening treatment is commonly applied to primarily enhance fatigue resistance. However, it can also improve the wear resistance due to the surface hardening it imparts and the induced dimples which may act as lubrication pockets [8]. 

One major characteristic of the SP process is its effect on the surface roughness of the material which tends to increase due to the impingement of the shots. This is not beneficial for both the material’s corrosion and tribocorrosion performance. Present investigations provide varying results with respect to the effect of the SP treatment on the corrosion performance. Studies have shown that it tends to enhance the corrosion resistance as the treatment results in grain refinement, thus increasing the grain boundary density which promotes the formation of the passive film [9]. Zhang et al. [9] investigated the effect that SP and ultrasonic shot peening treatments have on the corrosion resistance of SLM Ti64 in 3.5 wt % NaCl. The authors observed that both treatments enhanced the corrosion resistance however, in this regard the SP treatment was not as effective as the ultrasonic variety. This was attributed to the rougher surface generated by the conventional-SP treatment. A decrease in corrosion resistance due to the induced roughness was also observed by Zhan et al. [10] when investigating the effect different SP processes have on the corrosion resistance of S30432 steel in 3.5 wt % NaCl. The authors concluded that a single SP treatment was detrimental to the corrosion resistance in comparison with dual and triple SP treatments which cause limited roughening. Such a finding also suggests that the treatment parameters play an important role [11]. 

Currently, a limited number of investigations into the use of SP to enhance the tribocorrosion resistance of Ti-6Al-4V are available. Tribocorrosion is a mechanism whereby degradation occurs through corrosion in conjunction with wear. With respect to wear resistance, mixed behaviors have been observed when peened Ti64 was subject to linear reciprocating ball-on-flat testing in air. Whilst Tsuji et al. [12] observed a decrease in material loss for the shot-peened sample due to the surface hardening induced, Bansal et al. [13] concluded that the roughness induced was detrimental as it produced high coefficient of friction values. When investigating the tribocorrosion behavior of SP AISI 4140 low-alloy steel, Bozkurt et al. [11] concluded that the SP treatment resulted in a decrease in material loss rates with the maximum resistance being provided by the sample shot peened at the highest intensity equivalent to 24A. This was attributed to the increase in sub-grains and surface hardness induced by the treatment. However, the authors also noted that if the optimal Almen intensity is surpassed, then the resistance decreases due to increasing both the surface energy and roughness. 

Secondly, PVD coatings are commonly applied with the aim of enhancing both wear and corrosion resistance [14]. Coatings primarily increase the corrosion resistance of the substrate as they eliminate contact between the substrate and the corrosive medium [15]. Such coatings typically have high hardness values and are therefore not worn easily [15,16]. In turn, PVD-coated substrates typically exhibit an excellent corrosion and tribocorrosion behavior as observed by various investigations [17,18]. Furthermore, in this investigation, the coating applied has a multilayer construction. This is beneficial as compared to monolayer coatings, such coatings result in a dense structure with interfacial strengthening and have enhanced load carrying capabilities as the different layers aid to hinder the movement of dislocations [17]. Moreover, the various layers also prevent the propagation of the corrosive medium through any coating defects present in the substrate, leading to an overall good corrosion resistance [17,19,20]. This was confirmed by Çomaklı et al. [17] when investigating the corrosion-wear behavior of TiAlN/TiN multilayer and TiN and TiAlN monolayer coatings deposited onto Ti45Nb. The multilayer coated substrate obtained the lowest wear rate and coefficient of friction and exhibited the greatest resistance to corrosion. 

The effect that the coating has on tribological characteristics is also dependent on the coating’s adhesion to the substrate. The extent of adhesion determines the coating’s performance as it determines the load transferring abilities of the coating–substrate system. Poor adhesion causes the coating to easily flake off impacting negatively its function [21]. In the case of duplex treatment, shot peening induces a certain amount of surface roughness which may affect the coating adhesion. The high surface roughness increases the real contact area and thus, may encourage mechanical locking between the surface and the coating. However, the amount of roughness requires careful control as above a critical amount, this effect is reversed. Another reason for enhanced adhesion is due to the work hardening which increases the substrate’s resistance against deformation. Thus, the coating is better supported and its failure is delayed due to low shear stresses [22,23]. Zhang et al. [24] observed a decrease of nearly 50%, in the specific wear rate of duplex-treated, via high-energy shot-peening treatment and TiN coating, industrial pure titanium compared to the TiN coated-only sample. The duplex-treated sample also exhibited a lower coefficient of friction compared to the coated-only sample. 

Whilst the corrosion and corrosion-wear behavior of additively manufactured Ti-6Al-4V have been actively studied, presently the application of a duplex treatment to improve the corrosion and tribocorrosion performance of the additively manufactured substrate has not. The proposed duplex treatment is quite novel and the interaction of the peened surface and the PVD coating is not yet extensively studied especially with respect to the effect that such a combination has on the properties and characteristics of printed metallic materials. Thus, this investigation aims to shed light on the effect of this combination of treatments on the corrosion and tribocorrosion characteristics of additively manufactured Ti64 via testing in an artificial sea water solution. The results obtained provide an indication regarding the suitability of the proposed treatment applied to AM metallic components, allowing the faster production and replacement of parts employed in marine applications.

## 2. Materials and Methods

### 2.1. Substrate Material and Sample Preparation 

The cylindrical 20 mm diameter, 6 mm thick Ti-6Al-4V samples, having the composition listed in Table 1, were manufactured via selective laser melting (SLM) of a Ti-6Al-4V powder using an AmPro Innovations SP100 Metal 3D Printer (Suzhou, China) as per parameters listed in Table 2. The powder, supplied by Avimetal Powder Metallurgy Technology Co. Ltd. (Beijing, China), had a particle size ranging between 15 and 35 µm and composition listed in Table 1. The printed samples were subsequently heat treated using a TAV Dualjet TPH-200 (Lombardia, Italy) furnace, in a nitrogen atmosphere at 800 °C for 2 h followed by furnace cooling. The aim of this heat treatment is to transform the hard and brittle acicular α’ martensite phase formed upon printing and relieve induced thermal stresses. The 20 mm × 20 mm × 3 mm wrought mill-annealed Ti-6Al-4V samples, having the composition listed in Table 1, were supplied by Daido Steel Co. (Nagoya, Japan). The wrought and printed samples were ground and polished to a mean surface roughness, R_a_, of 0.006 µm and 0.049 µm, respectively.

### 2.2. Surface Treatments

The shot-peening treatment was carried out via an CBI Equipment Ltd. AB850 air blasting machine (Bournemouth, UK) at an Almen intensity of 0.20 mmA, 100% shot flow, 7 bar nozzle pressure using a nozzle of 80 mm length with 6 mm diameter and a nozzle-to-specimen distance of 100 mm. Zirshot Z300 ceramic shots were used having a diameter ranging from 300 to 435 µm. 

Coating deposition on the printed and SP-treated substrates was carried out using a Teer UDP800 (Beijing, China) closed field unbalanced magnetron sputtering ion plating system. Before deposition, any oxide layers were removed by subjecting the surface to sputter cleaning via high energy bombardment at 600 V and 0.5 A for 10 min followed by 10 min of cooling. The coating deposited has a multilayer structure composed of the following layers: Ti, TiN, TiAlN, and TiAlCuN. The coating was deposited at a bias of −90 V, target currents of 1 A for Cu and 8 A for Ti and Al, 35% optical emission monitor voltage, deposition pressure of 0.23 Pa and a target to sample distance of 145 mm. The designations used for the various sample conditions studied are explained in Table 3.

### 2.3. Surface and Near-Surface Characterization

Micrographic analysis of the surface and cross-section of the different sample conditions was carried out using a Carl Zeiss Axioscope 5 optical microscope, for low magnification analysis and a Carl Zeiss Merlin Gemini (Oberkochen, Germany) scanning electron microscope (SEM), for higher magnification analysis. Prior to imaging work, the substrates were etched using Kroll’s reagent composed of 5% HF, 13.5% HNO_3_ and 81.5% H_2_O. For chemical composition analysis, Ametek EDAX (Mahwah, NJ, USA) energy dispersive spectroscopy (EDS) analyzer was used in conjunction with the SEM. 

Surface roughness measurements were carried out via an AEP Technology NanoMap-500LS (Santa Clara, USA) contact profilometer. Scans were performed over 2500 µm at 25 µms^−1^ and a lateral resolution of 1 µm. Five scans were performed for each sample condition.

Using a Mitutoyo MVK-H2 (Kawasaki, Japan) microhardness tester in conjunction with a pyramidal diamond indenter, Vickers microhardness measurements were obtained. A load of 100 gf was applied for 10 s. A series of five indentations were carried out. 

### 2.4. Mechanical Testing

**Figure 1:**
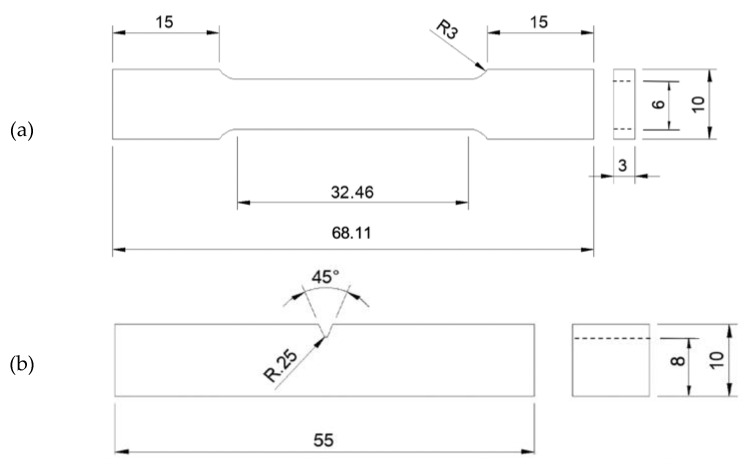
Dimensions of (**a**) tensile and (**b**) impact specimen. All units are in mm.

Tensile specimens having the geometry and dimensions as observed in Figure 1a, were tested using an Instron 5982 (Norwood, MA, USA) equipped with an Instron 2620-604 dynamic extensometer (USA). A strain rate of 3 mm min^−1^ was applied, and testing was carried out at a temperature of 25 °C and a humidity of 62%. The specimens were manufactured and tested as per ASTM E8/E8M-16—Standard Test Methods for Tension Testing of Metallic Materials. Charpy impact specimens with the geometry and dimensions shown Figure 1b were tested via an Instron 450MPX-J2 (USA) motorized pendulum impact testing system. The specimens were manufactured and tested as per ASTM E23-16—Standard Test Methods for Notched Bar Impact Testing of Metallic Materials. 

### 2.5. Corrosion Testing

Corrosion testing was carried out using a Gamry Interface 1000™ (Philadelphia, PA, USA) potentiostat connected to a 3-electrode setup. The working, counter and reference electrodes were the titanium-based test coupon, a platinum coated rod and a saturated calomel electrode (SCE) respectively. A surface area of approximately 0.785 cm^2^ was exposed to 300 mL of artificial seawater, formulated in accordance with ASTM D1141-98 (2021)—Standard Practice for the Preparation of Substitute Ocean Water. The solution was kept at a temperature of 25.0 ± 0.2 °C to simulate the marine environment as much as possible. Table 4 provides the chemical composition of the substitute ocean water. The stock solution was diluted in 300 mL deionized water in addition to 1.23 g of Na_2_SO_4_ and 7.36 g of NaCl. 

Initially, the open circuit potential (OCP) was monitored for 2 h to allow for its stabilization. This was followed by potentiodynamic polarization sweeps from a range of −0.2 mV versus OCP to +1.5 V versus reference at a sweep rate of 0.1667 mVs^−1^. The test was repeated three times for each of the sample conditions to ensure repeatability. To account for the effect of the increased surface roughness, the actual area of the surface-treated samples was calculated using the developed interfacial area ratio, S_dr_, obtained from height data obtained from profilometry. Following corrosion testing, the exposed areas were observed via optical and scanning electron microscopy.

### 2.6. Tribocorrosion Testing

For tribocorrosion testing, a Bruker UMT TriboLab (Billerica, MA, USA) set up with a reciprocating drive and a three-electrode tribocorrosion cell was used to analyze the corrosion-wear response of the samples. The cell was connected to a Gamry Interface 1000™ (Philadelphia, PA, USA) potentiostat to induce an anodic potential of 0.5 V with respect to the Ag/AgCl reference electrode. This value was determined from polarization curves obtained from potentiodynamic tests carried out. This potential ensures that all samples are in the passive regime when it is undergoing sliding wear.

The cell was filled with approximately 150 mL of artificial seawater formulated in accordance with ASTM D1141-98 (2021)—Standard Practice for the Preparation of Substitute Ocean Water. A 4.76 mm diameter Al_2_O_3_ counter-face was utilized. Alumina was chosen as it allows the sole characterization of the substrate due to its high hardness and inertness. A different ball for each test was utilized. The voltage was induced for 600 s without sliding followed by sliding for 2000 s at a load of 1 N (equivalent to a maximum calculated contact pressure of 678 MPa), frequency of 1 Hz and a stroke length of 3.5 mm. The load applied was selected after making calculations using Hertzian contact theory. This was determined by obtaining the load required to result in stress equivalent to the yield strength of untreated Ti64 (795 MPa). A value less than that acquired was chosen as the aim is to apply a contact pressure below the yield strength to avoid plastic deformation. The applied contact pressure in this investigation is approximately 15% less than the yield strength. 

During sliding, the dynamic anodic current and coefficient of friction values were recorded. Once sliding stopped, the anodic potential was monitored for a further 600 s. All tests were carried out at room temperature and repeated three times. 

Following testing, all wear tracks were analyzed via optical and scanning electron microscopy. Elemental analysis of any debris and artefacts present was also carried out. All wear track depths were measured via profilometry with three measurements taken for each wear track. From data collected during testing and measurements taken, the material loss rates were then quantified. The total wear and corrosion components can be quantified using the following Equations (1) and (2).

CW = C*+ W*(1)

where, CW is the total volumetric corrosion-wear rate (mm^3^s^−1^), W* is the rate of loss due to the mechanical wear component (mm^3^s^−1^) and C* is the rate of loss due to the corrosion component (mm^3^s^−1^). CW was obtained via measuring the total volume loss using the profilometer. C* was obtained via Faraday’s Law.

C*= IM/nFρ(2)

where, I is the current and equivalent to the area under the graph divided by the sliding duration (A), M is the atomic mass (gmol^−1^), n is the charge no. for the oxidation reaction (obtained from the passive region of their respective Pourbaix diagrams), F is Faraday’s constant (96,487 Cmol^−1^), ρ is the density of the substrate (gcm^−3^). 

## 3. Results and Discussion

### 3.1. Micrographic Analysis

**Figure 2:**
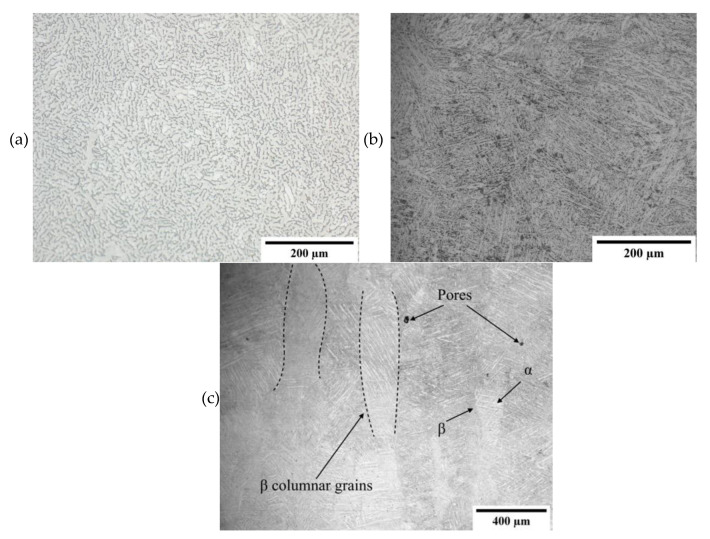
Low magnification micrographs of (**a**) surface of wrought Ti-6Al-4V, (**b**) surface of SLM Ti-6Al-4V, and (**c**) cross-section of SLM Ti-6Al-4V.

The wrought material has an equiaxed microstructure composed of the hexagonal α, the lighter phase, and cubic β; the darker phase as observed in Figure 2a. In comparison, the 3D printed sample following heat treatment, is composed from α + β phases forming a basket weave network in prior β columnar grains as observed in Figure 2b,c. This structure is typical for heat treatments carried out below the β transus temperature (980 °C, sub-transus), during which the initial acicular α’ martensite structure coarsens and transforms into α + β. The initial difference in microstructures is due to the rapid heating and cooling rates of the 3D printing process. The columnar grains are also a consequence of the process and form due to the epitaxial buildup of previously built layers which partially re-melt upon the formation of a new layer [25].

Following shot peening, the previously smooth surface of the printed sample loses its highly polished appearance due to the impact of the shots. On the microstructural level, the effect of grain refinement was not obvious. Moreover, the presence of twins was also not apparent which is a typical mechanism by which hcp crystals deform. Small initial grain size, peening intensity which does nott result in sufficient strain rates and high Al content which suppresses twin formation may all be contributing factors to the lack of twinning [26]. 

**Figure 3:**
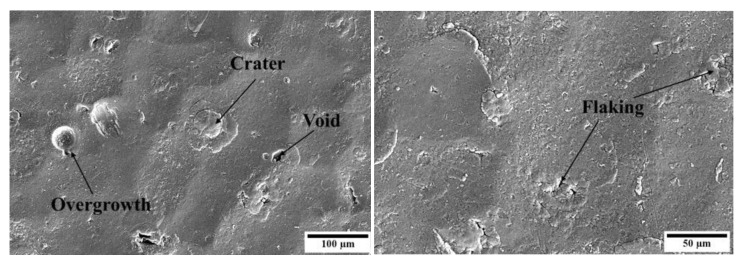
High-magnification micrographs of coating defects observed on the duplex-treated (DU) printed sample.

Upon the application of the coating, the underlying textured surface characteristic of SP could still be observed, no apparent levelling occurred. In addition, some artefacts typical of PVD coatings—including overgrowths, craters, and voids—were observed following coating deposition as observed in Figure 3. Overgrowths form due to the presence of surface features, such as asperities which act as nucleation sites on which a nodule composed of the coating grows. Craters form upon detachment of such overgrowths. Present voids are most likely shallow depressions or pits due to the shadowing effect, coating growth at the walls is slower compared to at the surface. Flaking observed could be attributed to the high compressive stresses induced upon deposition or due to the thermal stresses induced upon cooling [27]. Overall, the presence of defects was expected due to the rough nature of the underlying substrate preventing the formation of a perfectly uniform layer.

**Figure 4:**
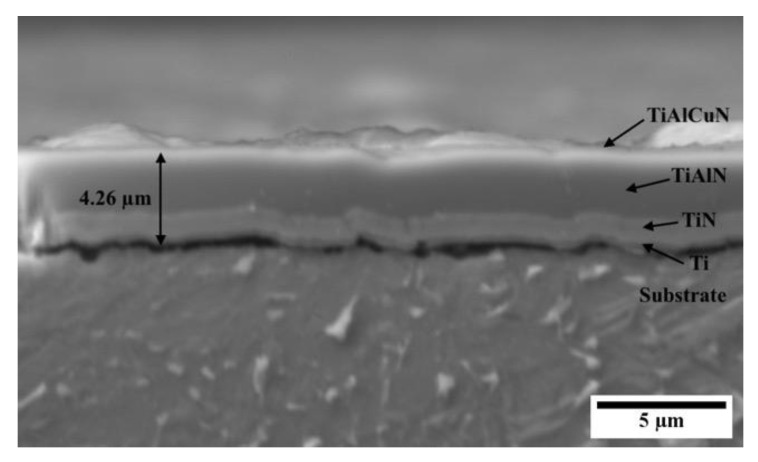
High-magnification micrograph of the cross-sectioned PVD coating deposited on the duplex-treated (DU) printed sample.

Further analysis of the coating’s cross-section was carried out. The coating shown in Figure 4 was observed to have an average total thickness of 4.26 µm comprising the various layers visible. Due to the multilayer structure, the columnar growth is continuously interrupted enhancing the structural density of the coating and decreasing the grain size [17]. Such a dense coating is likely to protect the underlying substrate from corrosive media as the multiple layers hinder its propagation towards the bulk.

### 3.2. Surface Topography Analysis

Roughness measurements have been tabulated in Table 5. Compared to the wrought sample (W), the printed and polished (P) samples demonstrated a significantly higher R_a_ value due to retained porosity after polishing. However, compared to the initial as-printed condition, the polishing process resulted in a significant decrease in the surface roughness [28]. The R_a_ and R_z_ roughness values were the highest for the printed and shot-peened (SP) and duplex (DU)-treated samples. This was expected due to the roughening effect imparted by the SP treatment. The impact of shots results in sharp protrusions which in turn depend on the peening intensity. The maximum depth obtained is dependent on the shot diameter used [9]. Furthermore, the deposited coating did not enhance the surface finish. This is most likely due to the limited number of layers, thus the thicknesses are insufficient to cause smoothing or result in levelling. Jiang and Arnell [29] observed the opposite effect; however, it was observed that, due to levelling, the coating at asperities is thinner thus promoting crack initiation and increasing the susceptibility of the coating to failure via fragmentation since the layers are not evenly distributed. 

### 3.3. Hardness Analysis

The Vickers microhardness measurements obtained for the various samples are listed in Table 6. In literature, as-printed Ti-6Al-4V, which has not undergone any heat treatment, is reported to have a hardness as high as 409 HV due to the acicular α’ martensite which is a hard and brittle phase [25,30]. This high hardness is then observed to decrease upon the application of a heat treatment depending on the heat treatment temperature [25]. In this investigation, the printed sample was heat treated and a hardness of around 330 HV_0.1_ was recorded. Following SP, the hardness of the printed sample increased by 13% confirming that a degree of work hardening occurred. A similar increase in magnitude was observed by Zhang et al. [9] for SP SLM Ti64. Such hardening is due to grain refinement, which results in an increase in grain boundaries and thus a greater hindrance to dislocation movement and induced compressive residual stresses. 

**Figure 5:**
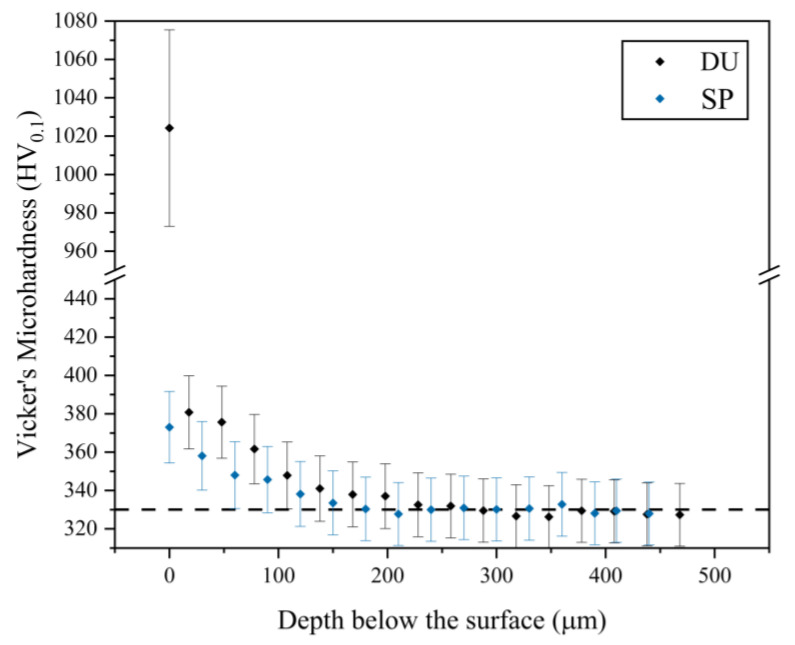
Plot of profile microhardness measurement for the printed and shot-peened sample (SP) and the printed and duplex-treated sample (DU).

The effect of the treatment was further confirmed by the profile hardness measurements. The hardness values were highest at the surface and decreased gradually to the bulk hardness resulting in an affected depth of around 180 µm as observed in Figure 5. Depths of similar magnitudes were observed by other authors [9,31]. The duplex treatment provided an increase in surface hardness of 210% over the hardness of the 3D printed sample. High hardness values for such coatings are expected; moreso in this case, since the multilayer structure hinders dislocation movement, thus enhancing the load-carrying capabilities of the coating [17]. Compared to a multilayer Ti/TiN/TiAlN coating deposited onto a carbide substrate studied by Vereschaka et al. [32], where values ranging between 1764 and 2471 HV_0.05_ were recorded, lower values were recorded in the current investigation. This is most likely due to the softer Ti64 substrate which contributes to this diminished value as the indentation depth is most likely greater than 10% of the coating thickness when obtaining microhardness measurements.

### 3.4. Mechanical Testing

#### 3.4.1. Tensile Testing

Table 7 presents the results obtained following tensile testing of the printed and heat-treated specimens, in comparison with values observed in literature for wrought and printed and heat-treated Ti64. For the latter, values listed were from investigations where a similar heat treatment as this investigation was applied. 

As observed, values obtained are within the range of values observed in literature. However, the elongation obtained for the printed and heat-treated samples is on the lower end of the range. Typically, larger elongation values are observed following heat-treatment as the brittle martensitic phase is transformed into alpha and beta phases [35,36,37]. Furthermore, the 3D printed material is observed to have properties comparable to the wrought material, except for the elongation. This is a commonly observed difference, and it is likely attributed to the different microstructures obtained by the additive manufacturing and conventional manufacturing routes, where the latter includes processes such as forging, casting, and rolling [33]. 

In this investigation, it appears that the chosen HT temperature did not impact the ductility significantly. This may be due to defects such as micro-cracks, lack of fusion and pores which the 3D printing process is prone to, and which also affect the mechanical properties as they act as stress concentrators [38,39]. Plessis et al. [39] noted that 1% porosity had a significant effect on the mechanical properties in cases where printing parameters were not optimal resulting in lack of fusion defects which are large and irregularly shaped. 

Furthermore, the build orientation also plays a significant role. In this investigation, the specimens were horizontally built, thus a relatively low elongation was expected since—during tensile testing—tension is applied perpendicular to the columnar grains. Therefore, the number of effective grain boundaries which can hinder crack growth is decreased when samples are horizontally built [35].

#### 3.4.2. Impact Testing

Compared to typical values observed in literature, the specimens exhibited a very good impact performance since the typical impact energy values vary between 4 J and 10 J for the as-printed condition. Such low values are due to the high brittle martensitic phase fraction [40,41,42,43,44]. In this investigation, for printed and heat-treated Ti64, a total energy value of approximately 14 J was observed. 

Compared to wrought samples, a reduced impact energy was obtained since for the former values up to 49 J are typically observed [45]. However, this value is heavily dependent on the microstructure, and for additively manufactured specimens it also depends on the residual stresses present. Lee et al. [42] noted that for Ti64 a wide variation of energy values exists, depending on the volume fraction of the phases.

**Figure 6:**
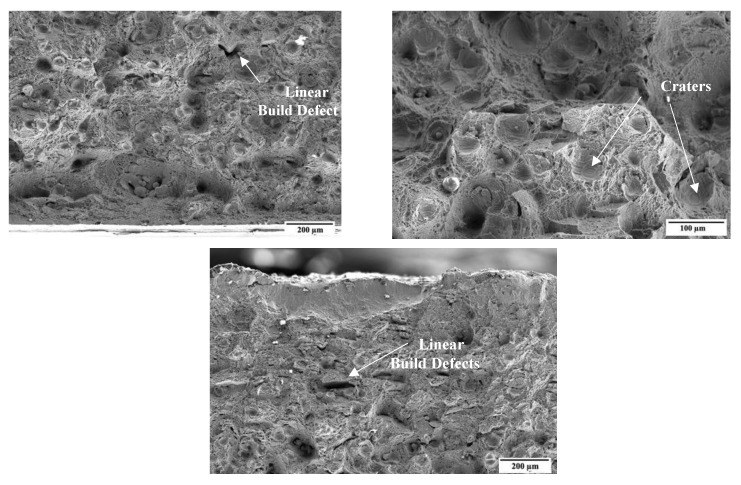
High magnification micrographs of the fractured heat-treated specimen.

Following testing, the fractured surfaces were analyzed using a scanning electron microscope to determine the type of fracture present. Figure 6 depicts high magnification micrographs obtained of the fractured surface of the printed and heat-treated specimen. Visually, the fracture appears to be brittle as there was no considerable plastic deformation. Cleavage facets can be observed which are a characteristic of brittle fracture. However, some dimples can also be observed which are a typical characteristic of ductile fracture. Thus, the fracture type is most likely mixed-fracture, as observed by Kazachenok et al. [45] for SLM Ti-6Al-4V.

This decrease in toughness for the printed samples compared to the wrought, may also be related to defects such as pores and presence of impurities which the printing process is prone to [40,41]. In Figure 6, the presence of pores, solid un-melted powder particles and other build defects are highlighted. Several linear building defects can be observed. Similar characteristics were observed by Wu et al. [44]. Such defects indicate that the build quality is not consistent from one layer to another. These were observed to negatively impact the load bearing capacity resulting in a decreased toughness. Furthermore, a significant number of crater-like defects or larger dimples can be observed having features of cleavage fracture inside. These appear to represent the occurrence of particle detachment or pull-out.

### 3.5. Corrosion Testing

**Figure 7:**
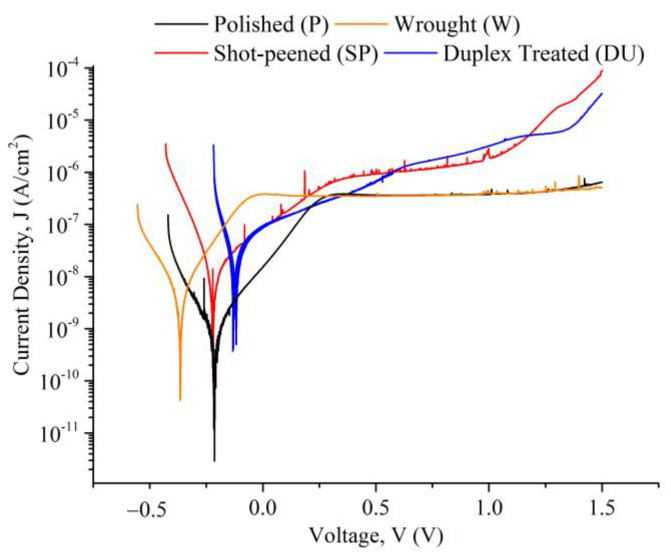
Potentiodynamic polarization curves obtained for each of the sample conditions tested in a simulated ocean water solution.

**Figure 8:**
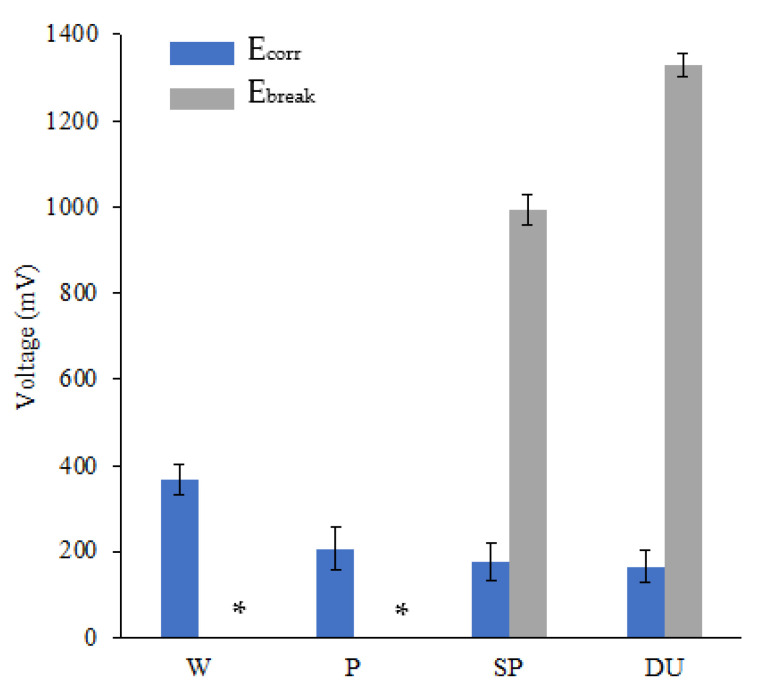
Plot of E_corr_ and E_break_ values for the different sample conditions. * For the wrought (W) and printed and polished (P) sample, the E_break_ value is equal to 0 as for both conditions, this phenomenon was not observed.

**Figure 9:**
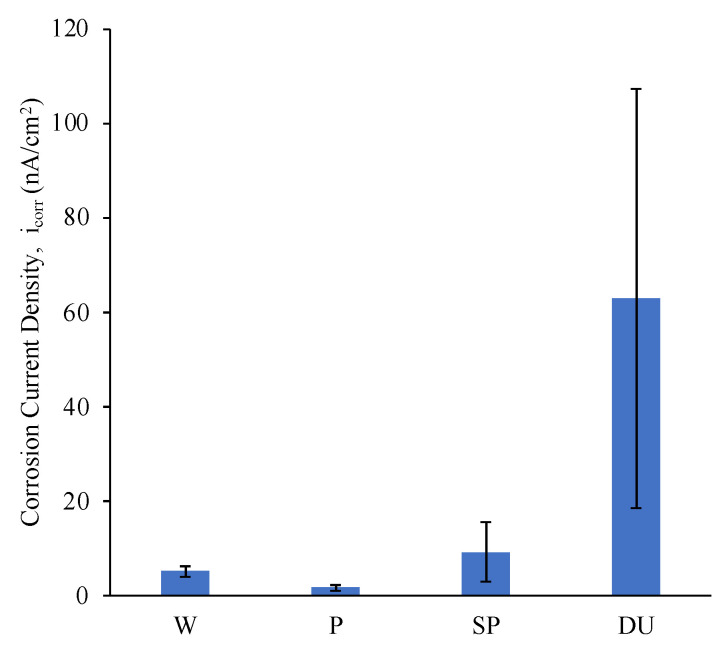
Plot of i_corr_ values for the different sample conditions.

**Figure 10:**
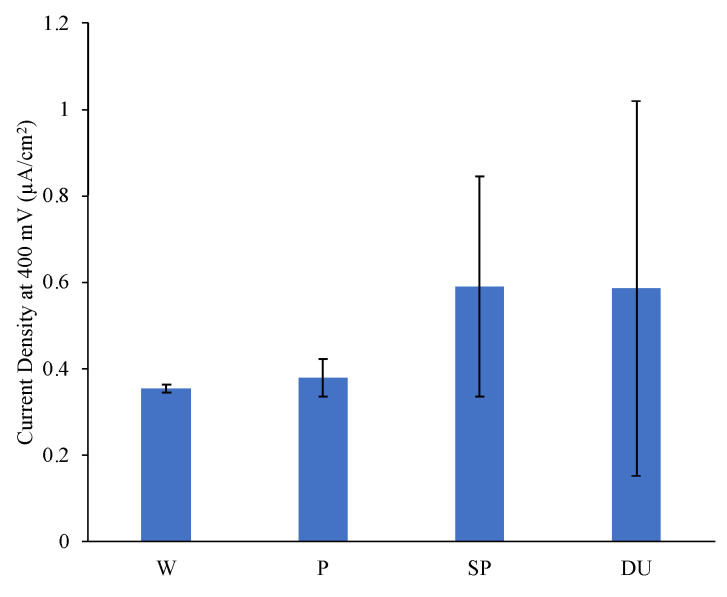
Plot of current density values at 400 mV for the different sample conditions.

Representative potentiodynamic (PD) curves obtained from the three repeats carried out for all sample conditions tested in simulated ocean water can be observed in Figure 7. For comparison bar graphs of the E_corr_ and E_break_, i_corr_ and passive current density at 400 mV vs SCE values were plotted as observed in Figure 8, Figure 9 and Figure 10. The plots depict the range of the three repeats. 

From the PD curves and as observed from the E_corr_, i_corr_ and passive current density at 400 mV vs SCE values (Figure 8, Figure 9 and Figure 10), the printed and polished Ti-6Al-4V samples exhibit a slightly better corrosion performance compared to the wrought samples exhibited through the more positive E_corr_ and smaller current density values obtained. On the other hand, compared to the printed and polished sample, the shot-peening and duplex treatment—which were applied to printed Ti64 only—resulted in similar E_corr_ values; however, a greater i_corr_ than the polished sample was recorded for both conditions. 

Previous investigations observed no change or a decrease in corrosion resistance following shot peening when testing in 3.5 wt % NaCl, 0.9 wt % NaCl and Ringer’s solutions [9,46,47]. This lack of improvement in corrosion resistance can be correlated to the high surface roughness as it increases the interaction area [9,48]. Furthermore, as observed in Figure 7, for the shot-peened and duplex-treated samples a steep increase in current values was exhibited at around 1000 mV and 1300 mV, respectively. This value is regarded as the pitting potential, E_break_ or E_pit_ and was observed for all three repeats (Figure 8). It indicates that, at these potentials, pits are starting to form. On the other hand, the wrought and the printed sample do not exhibit this phenomenon, most likely due to having a more stable passive film which was less likely to breakdown [49]. The high surface roughness induced by the peening treatment of the shot-peened (SP), compared to that of the wrought (W) and printed and polished sample (P), is likely contributing to the formation of an nonuniform and unstable passive film with more active sites that encourage its breakdown, as evidenced by the pitting observed [9]. The breakdown of the film occurs as chloride anions adsorb at defective sites reacting with the TiO_2_ passive film causing it to weaken and forms soluble high-energy coordination complexes [50]. 

Furthermore, shot peening induces dislocations as well as stress. Whilst some authors observed that induced dislocations promoted passive film formation [9], others observed an opposite effect. Buhagiar and Dong [51], in their investigation into the corrosion performance of low temperature carburized austenitic stainless steel in Ringer’s solution, observed that the treatment applied generated a significant number of dislocations, similar to shot peening. The authors observed that such dislocations caused the passive film to rupture exposing the underlying substrate to the solution. This led to the anodic dissolution of the exposed site prior to the re-passivation of the film, resulting in crevice and pitting corrosion conditions. 

**Figure 11:**
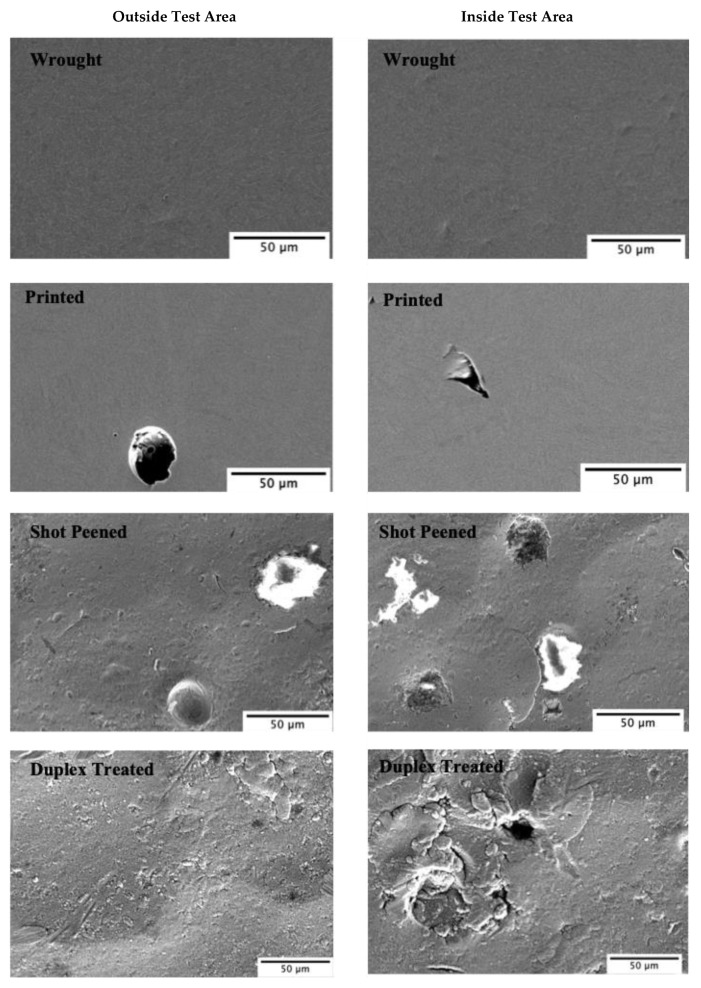
High-magnification images of outside and inside the corrosion test area for all sample conditions.

**Figure 12:**
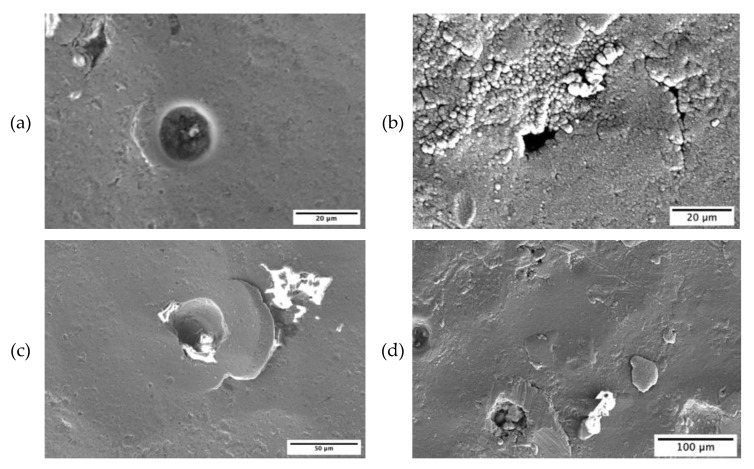
High magnification micrographs obtained post corrosion testing in simulated ocean water, of (**a**) a pit and (**c**) surface damage in the form of cracking and craters, observed on the shot-peened (SP) sample, (**b**) a pit and (**d**) coating defects including cracks and delamination, observed on the duplex-treated (DU) sample.

To better understand why poor corrosion resistance was observed for the shot-peened (SP) and the duplex-treated (DU) samples, the samples were observed using a scanning electron microscope. As observed in Figure 11, for the wrought (W) and printed and polished (P) samples negligible corrosion damage was observed. However, as seen in Figure 11 and Figure 12, for both the shot-peened (SP) and duplex-treated (DU) samples, what appear to be pits were observed. Craters and cracks observed on the shot-peened sample’s surface are attributed to damage sustained during the application of the peening treatment. Such defects allow the solution to penetrate and become trapped, resulting in a more corrosive environment thus increasing the probability of localized corrosion to take place. For the duplex-treated sample, several defects—such as cracks and delamination—were observed. Additionally, in the test area several pits formed in the coating deposited could be seen. Thus, its poor performance similar to that of the shot-peened sample is attributed to such defects which allow the propagation of the corrosive medium towards the substrate enhancing the corrosion under the coating rendering the coating unprotective. 

The formation of such defects is also linked to the high roughness of the underlying substrate. In literature, as the underlying substrate’s surface roughness is increased, the corrosion protection provided by the coating is observed to decrease [52,53]. As PVD coatings are deposited, their nucleation and growth are dependent on the surface topography. A smooth surface promotes a lower defect density and improves adhesion. In comparison, a rougher surface encourages the formation of porosity and growth defects. This results in a local loss of adhesion and increases the coating’s permeability, decreasing its corrosion-barrier performance [53].

The corrosion current density, i_corr_, provides a good indication regarding the corrosion resistance of the samples. A smaller corrosion current density value indicates that the surface has a higher affinity to passivation and a lower corrosion rate [49]. From Figure 10, it was observed that at around 400 mV, where the samples are all in the passive regime, all conditions exhibit a similar corrosion behaviour, thus confirming that at this point the application of the surface treatments neither increased nor decreased the corrosion resistance of the material.

### 3.6. Tribocorrosion Testing 

#### 3.6.1. Dynamic Current and COF Measurements

**Figure 13:**
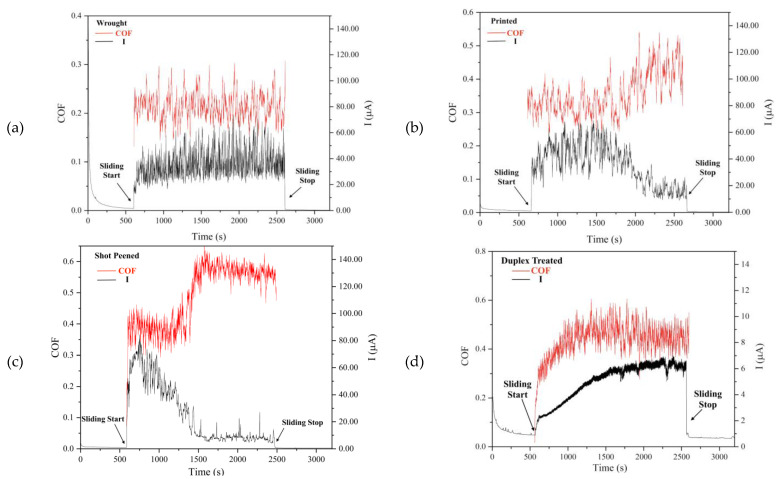
Dynamic anodic current and COF plots (**a**) wrought, (**b**) printed and polished, (**c**) printed + shot-peened, and (**d**) printed + duplex-treated Ti-6Al-4V. The plot in (**d**) has a current y-axis scale that is 10 times smaller in magnitude compared to the y-axis scale in (**a**–**c**).

As observed in Figure 13, for all sample conditions upon sliding, an increase in anodic current was observed which dropped back to initial values once sliding stopped. This increase is due to the removal of the passive film exposing the underlying substrate to oxidation [54,55]. The anodic current and coefficient of friction (COF) measurements recorded against time can be observed in Figure 10. The current values recorded for the polished and printed (P) sample dropped from currents ranging from 40 to 70 µA to currents ranging from 10 to 25 µA (Figure 13a). Moreover, the printed and shot-peened (SP) sample also exhibited a similar behavior to the P sample, whereby the anodic current and COF exhibited an inversely proportional relationship. The decrease in current implies a decrease in the amount of new substrate exposed to the electrolyte and it is attributed to the formation of oxidized patches which provide this twofold effect [55]. 

For the P sample, the COF increased from 0.30 to 0.47, similar to values observed in literature [55]. Initial low values could be due to smoothing of the sliding surfaces, values then increase due to micro-fragmentation and adhesive wear resulting in a wear track which is no longer smooth [56]. Furthermore, for the SP sample several spikes, once current values stabilized at lower values, were also observed (Figure 13b). Such spikes are related to the development of wear particles where their formation results in de-passivation and subsequent re-passivation of the substrate. Once the particle is ejected or crushed, the COF increases whilst the current decreases again [57]. This increase in COF may also be related to the formation of asperities which decrease the contact area; thus, the contact pressure at such areas increases, resulting in roughening.

For the SP samples, currents as high as 80 µA were recorded suggesting that the substrate was immediately exposed hence confirming further that the SP treatment was ineffective. The high COF values observed are typical for SP-treated Ti64 since the treatment increases the surface roughness. This increased roughness was most likely detrimental to the tribocorrosion behavior [58].

For the printed and duplex-treated sample (DU) sample, low current values around 6 µA were recorded which are typical for PVD coatings subjected to such testing mostly due to their high hardness [17]. Both the COF and current are observed to increase with time (Figure 13c) as wear occurs gradually at a diminished rate until the coating undergoes complete failure. The coatings can undergo three different wear-corrosion mechanisms [59]:

- Type I: the removal or damage of the passive layer present at the coating surface and its subsequent re-passivation.
- Type II: the substrate undergoes galvanic attack causing the coating to blister or become completely removed during sliding.
- Type III: the counter-body undergoes galvanic attack causing it to roughen resulting in abrasive wear.

The DU sample underwent Type I corrosion-wear. Type I is also applicable to the uncoated substrates (W, P, and SP) and is reflected in the constant fluctuations of corrosion-current, corresponding to the continuous re-passivation and de-passivation, as observed in Figure 13a–c. Moreover, from the low currents obtained it can be noted that the coated was effective in reducing the Type I corrosion-wear significantly. Furthermore, no blistering of the coating was observed thus Type II corrosion-wear could not have occurred and Type III corrosion-wear was not possible since an alumina ball was used. Thus, no galvanic coupling could ensue. A COF value of 0.42 was obtained, a high COF value was expected due to its deposition on a previously shot-peened surface. In previous investigations, this increased roughness has been observed to be detrimental to the corrosion-wear resistance of the treated sample, negatively impacting the coating’s adhesion and wear resistance [60,61]. However, despite the high COF values, very low anodic current values were recorded reflecting the effectiveness of the duplex treatment which is attributed to the multilayer structure of the coating. Such coatings result in a small increase in corrosion current over time due to: (i) their lower tendency to dissolution accredited to their high chemical stability; or (ii) the small ratio of the active to passive regions in the track [62]. A diminished active area is obtained due to the high hardness of the coating which results in a reduced wear track width. 

#### 3.6.2. Material Losses

**Figure 14:**
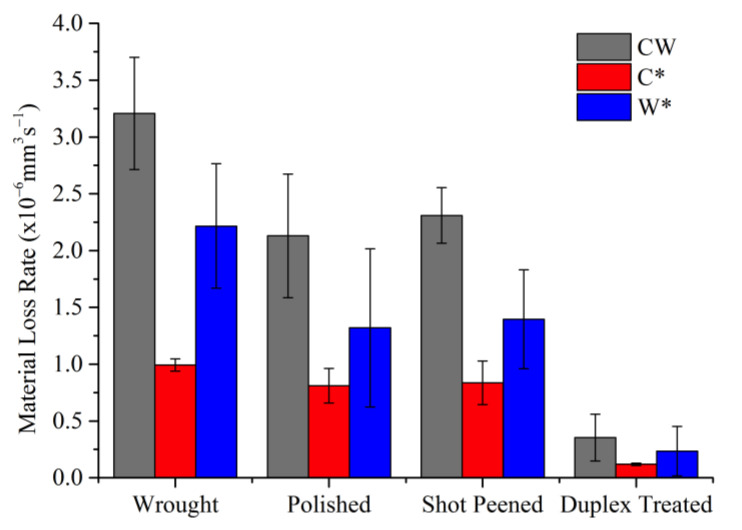
Plot of material losses obtained from testing the sample conditions over a sliding duration of 2000 s at a load of 1 N and frequency of 1 Hz against an alumina counter face in simulated ocean water.

**Figure 15:**
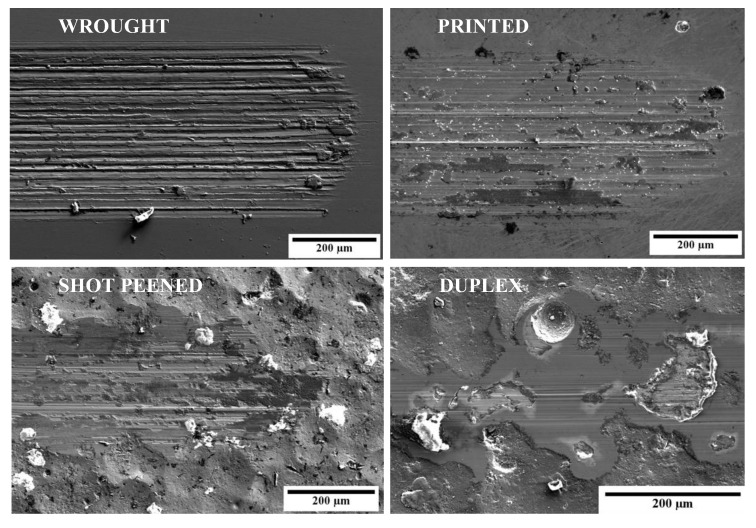
High-magnification micrographs of the wear tracks obtained for the various sample conditions.

Figure 14 provides the corrosion-wear data calculated from the anodic current and wear track volume measurements. The data confirm that the DU sample has superior corrosion-wear resistance, showing a reduction in rate of material loss by almost a factor of 6 compared to the P and SP samples. Thus, the treatment was effective mainly due to the increased hardness and corrosion resistance provided by the multilayer coating [55,60]. No significant difference between the corrosion-wear resistance of the W and P sample is present however the slight improvement, observed by the lower current values recorded, of the P sample is attributed to the oxidized patches formed, as observed via optical imaging (Figure 15), and analyzed via EDS, which decreasee the contact between the sample surface and the solution. The SP treatment also appears to be ineffective as the resistance did not improve relative to the P sample. The increased roughness rather enhances the degradation mechanisms as on average greater material loss due to mechanical wear (W*) is observed compared to the P sample.

From the material losses, the ratio of loss due to the corrosion component and loss due to the wear component (C*/W*) provides an insight in the dominant degradation mechanisms present [55,63]. The ratios obtained can be observed in Table 8. Since all values are between 0.1 and 1, this suggests that synergism is present, and the most dominant mechanism is wear-corrosion. Therefore, the greatest losses are caused by mechanical wear with limited contribution from corrosion which is also reflected in the results obtained. The former contributed to 69%, 62%, 63%, and 66% of the corrosion-wear for the W, P, SP, and DU samples respectively.

The wear tracks obtained for the printed (P), printed and shot-peened (SP), and printed and duplex-treated (DU) samples are observed in Figure 15. Grooves parallel to the direction of motion are observed for all sample conditions and are evidence of abrasive wear. For the DU sample, these grooves are distinctively shallow confirming its increased wear resistance. Both the P and SP samples show signs of oxidation in the form of patches all over the wear track. These were confirmed to be a build-up of TiO_2_ by EDS analysis and a result of adhesive wear. Strong adhesion is experienced between the opposing surfaces at asperities resulting in high frictional forces. As these asperities are removed, debris is generated which is then compacted resulting in patches which protect the underlying substrate. The patches may result in an increase in COF values however they also aid with load carrying due to their higher hardness. If not compacted, the debris freely moves, forming grooves and scratches by abrasion [55,60]. The occurrence of adhesive wear was also evidenced by the unstable COF and high wear rates of the P and SP samples [55].

When comparing the P and SP sample wear tracks, no significant difference can be observed which is opposed to that observed by Ganesh et al. [7] when subjecting wrought SP Ti64 to a pin-on-disc wear test against a hardened steel counter-body. The authors observed thicker and coarser grooves in the wear track of the treated material, which suggests that it exhibited a greater resistance to abrasive wear due to the higher hardness of the material. It appears that the limited increased hardness due to the peening treatment was not sufficient to hinder material removal as opposed to the duplex-treated sample. 

For the DU-treated substrate, minimal wear was observed, and this mainly occurred at asperities as a non-uniform track was obtained. At certain areas, significant material removal occurred likely due to the high contact forces experienced at asperities. However, this was not accompanied with a significant increase in current, as the ball did not wear the coating enough such that contact was made with the exposed underlying substrate. The exposed substrate was then protected by the passive layer. Furthermore, no blistering was observed, thus confirming that the coating underwent Type I corrosion-wear. Despite the improvement, compared to other coated substrates tested under similar conditions degradation endured in the current investigation was more significant [24,55,64]. A contributing factor may be due to the high surface roughness which appears to be aggravating the wear mechanisms present locally. However, the overall reduced wear endured by the DU samples is also evidenced by the decreased wear track depths obtained as observed in Table 8. 

## 4. Conclusions

Mechanical, corrosion, and tribocorrosion testing were carried out on additive manufactured Ti-6Al-4V alloy which was surface treated by shot peening and a duplex treatment, consisting of shot peening and a TiAlCuN coating deposited by physical vapor deposition. The following conclusions were drawn from the results obtained:

- The shot-peened and duplex-treated samples showed an increase in the *R_a_* and *R_z_* roughness by a magnitude of 30 and 8 respectively, while the surface hardness increased by 13% and 210%, respectively. The hardened depth was equivalent to around 180 µm.
- The printed and heat-treated material was observed to have comparable mechanical properties to the wrought material, except for the elongation. It also exhibited a decreased impact energy absorbed compared to wrought. In both cases, this was attributed to the different microstructures resulting from the different manufacturing processes and printing process defects.
- The printed and polished sample was observed to have a better corrosion resistance than the wrought, when testing in simulated ocean water at a temperature of 25.0 ± 0.2 °C.
- Both surface treatments were ineffective in improving the corrosion performance of the printed sample. This was attributed to the high surface roughness induced by the peening process. For the shot-peened sample, it resulted in a non-uniform passive film prone to corrosion attack. For the duplex-treated sample, it increased the coating’s defect density, making the substrate more susceptible to pitting and crevice corrosion.
- The corrosion-wear resistance increased in the following sequence, wrought < polished ≈ shot peened < duplex. The excellent performance of the duplex-treated sample is credited to the increased surface hardness and to the barrier protection provided by the coating which diminished Type-I corrosion-wear significantly.
- The similar behavior of the printed and shot-peened sample indicates that the peening treatment was ineffective in enhancing the corrosion-wear resistance, as it does not protect the substrate from Type-I corrosion-wear.

From the results obtained, it can be concluded that printed Ti64 can be used in place of wrought Ti64 in applications where high strength is required. With further printing and heat treatment parameter optimization, a perfect combination of mechanical and impact properties can be achieved. The proposed duplex treatment proved to be beneficial in applications where the component is under friction conditions in a corrosive environment as experienced by components used in the marine transportation industry.

## Figures and Tables

**Table 1:** Chemical composition of 3D printed and wrought Ti-6Al-4V samples.

	Ti	Al	V	Fe	O	N
Ti-6Al-4V powder (wt %)	Bal.	6.13	4.00	0.16	0.09	0.01
3D Printed (wt %)	Bal.	7.24	2.89	/	/	/
Wrought (wt %)	Bal.	6.03	4.19	0.20	/	/

**Table 2:** 3D printing parameters.

Parameter	Value
Laser Power	100 W
Laser Speed	600 mms^−1^
Beam Offset	10 µm
Layer Thickness	30 µm
Hatch Distance	70 µm
Internal Overlap	≥100 µm
Hatch Type	Stripe
Stripe Width	10 mm
Rotate Each Layer	Start Angle: 45°, Angle Change: 66.66°

**Table 3:** Designations used for the various sample conditions.

Designation	Sample Condition
Wrought (W)	Conventionally manufactured mill-annealed Ti-6Al-4V
Printed (P)	SLM + heat-treated + polished Ti-6Al-4V
Shot-peened (SP)	SLM + heat-treated + polished + shot-peened Ti-6Al-4V
Duplex-treated (DU)	SLM + heat-treated + polished + shot-peened + coated Ti-6Al-4V

**Table 4:** Chemical composition of stock solutions.

Chemical Compound	Concentration (gL^−1^)
Stock Solution No. 1	
MgCl_2_.6H_2_O	555.6
CaCl_2_	57.9
SrCl_2_. 6H_2_O	2.1
Stock Solution No. 2	
KCl	69.5
NaHCO_3_	20.1
KBr	10.0
H_3_BO_3_	2.7
NaF	0.3

**Table 5:** Roughness measurements obtained for all sample conditions.

Sample	Arithmetic Mean Roughness R_a_ (µm)	Mean Roughness Dept R_z_ (µm)
W	0.006 ± 0.001	0.072 ± 0.024
P	0.049 ± 0.014	1.325 ± 0.518
SP	1.470 ± 0.126	11.592 ± 0.950
DU	1.548 ± 0.294	10.781 ± 2.088

**Table 6:** Surface hardness measurements.

Sample	Hardness (HV_0.1_)
W	348 ± 18
P	330 ± 3
SP	375 ± 5
DU	1024 ± 58

**Table 7:** Results obtained from tensile testing and values quoted in literature for the mechanical properties of wrought Ti-6Al-4V.

Sample	UTS (MPa)	YS (MPa)	Elongation (%)
Printed + HT	873 ± 25	804 ± 20	6 ± 1
Printed + HT (Literature) [33]	837–1176	768–1104	5–13
Wrought Ti-6Al-4V [34]	900	830	14

**Table 8:** Wear track depths and C*/W* ratios for the different sample conditions.

Sample	Wear Track Depth (µm)	Ratio of Loss due to the Corrosion Component and Wear Component (C*/W*)
W	7.24 ± 0.54	0.45
P	5.38 ± 0.87	0.61
SP	6.42 ± 0.92	0.60
DU	2.84 ± 0.49	0.50

## Data Availability

Not applicable.
